# Hyperbaric oxygen pretreatment on endothelial cell injury via heat shock factor 1 in decompression sickness

**DOI:** 10.3389/fmolb.2025.1617318

**Published:** 2025-06-13

**Authors:** Caiyi Xu, Quan Zhou, Xiangyang Meng, Jiahe Zhou, Xuhua Yu, Juan Zheng, Hongjie Yi, Guoyang Huang, Weigang Xu

**Affiliations:** ^1^ Naval Medical Center, Naval Medical University, Shanghai, China; ^2^ The First Affiliated Hospital, Naval Medical University, Shanghai, China

**Keywords:** decompression sickness, hyperbaric oxygen, heat shock factor 1, pulmonary microvascular endothelial cells, protective protein

## Abstract

**Background:**

This study investigated the protective role and mechanisms of hyperbaric oxygen (HBO) pretreatment in enhancing vascular endothelial cell (VEC) resistance to bubble-induced injury by focusing on heat shock factor 1 (HSF-1) to prevent decompression sickness (DCS).

**Methods:**

Primary cultured rat pulmonary microvascular endothelial cells (PMVECs) and Sprague–Dawley rats were used as experimental models. Western blot analysis was performed to examine the activation patterns of HSF-1 and the expression of downstream proteins after HBO exposure. Specific inhibitors were used to delineate the signaling pathways involved in HSF-1 activation. At peak protein expression time points, a cellular bubble-induced injury model and a rat DCS model were established. The functional roles of HSF-1 and its downstream proteins in protection against HBO were evaluated using specific inhibitors and siRNAs.

**Results:**

HBO significantly enhanced the nuclear translocation of HSF-1 and upregulated the expression of downstream heat shock proteins 27 and 40 in PMVECs and lung tissues, peaking at 12 and 18 h after HBO exposure, respectively. The reactive oxygen species (ROS) levels and phosphorylation of ERK1/2, P38 MAPK, and AKT were markedly elevated after HBO exposure. AKT inhibition substantially suppressed HSF-1 activation, whereas ERK1/2 and P38 MAPK inhibition had no effect. ROS scavenging with Mito-Tempo reduced AKT phosphorylation and HSF-1 activation. Bubble-induced injury significantly decreased the viability of PMVECs and increased the levels of endothelial microparticles, inflammatory cytokines, and endothelial injury markers in culture media. HBO pretreatment ameliorated these pathological changes, which were reversed by HSF-1, HSP27, or HSP40 inhibition. In DCS rats, HBO pretreatment decreased lung wet-to-dry ratios, histopathological scores, serum inflammatory cytokines, endothelial microparticles, and endothelial injury markers. These benefits were reversed by the HSF-1 inhibitor KRIBB11.

**Conclusion:**

HBO pretreatment enhanced VEC resistance to bubble injury and mitigated DCS-related damage in rats via HSP27 and HSP40 expression upregulated by the ROS/AKT/HSF-1 signaling pathway.

## Background

Decompression sickness (DCS), arising from the release of dissolved inert gases following abrupt reductions in ambient pressure, poses a critical health risk to divers and individuals exposed to hyperbaric environments. Overall rates of DCS per dive are 0.004%–0.01% among recreational divers, 0.030% in U.S. Navy divers, and 0.095% in commercial divers (Vann et al., 2011; Mahon and Regis, 2014; Mitchell et al., 2022). DCS is characterized by sudden onset, severe progression, and challenging treatment, underscoring the critical importance of prevention.

Vascular endothelial cell (VEC) injury induced by intravascular bubbles plays a pivotal role in the pathogenesis of DCS (Pontier et al., 2009). These bubbles directly interact with VECs, causing mechanical damage, and further exacerbate injury through the activation of inflammation, coagulation, and apoptosis (Åsmul et al., 2017). VECs are multifunctional cells that not only maintain the blood–tissue barrier but also govern vascular tone, control substance exchange, and modulate inflammatory and immune processes (Šegrt Ribičić et al., 2019; Mahon and Regis, 2014). The bubble-induced VEC damage disrupts these essential physiological functions driving the initiation and progression of DCS (Šegrt Ribičić et al., 2019; Zhang et al., 2016). A series of studies by our group focused on the relationship between VEC and DCS. In rat and pig DCS models, significant VEC injury was observed, which correlates positively with the volume of circulating bubbles (Zhang et al., 2016; Qing et al., 2017). Furthermore, the administration of VEC protectants such as escin and edaravone not only alleviates VEC injury but also significantly reduces the incidence of DCS and secondary biochemical injuries (Qing et al., 2017; H et al., 2013). These findings suggest that preserving VEC integrity is a promising strategy for preventing DCS.

Hyperbaric oxygen (HBO) is a definitive intervention for DCS treatment. By breathing pure oxygen at pressures exceeding one atmospheric absolute, HBO exerts protective effects by promoting inert gas elimination, reducing intra-tissue bubble volume, and enhancing tissue oxygenation (Pontier et al., 2009; Schottlender et al., 2021). Additionally, HBO can also induces moderate oxidative stress via increasing the intracellular ROS level, which mobilizes endogenous protective mechanisms by activating the expression of protective proteins such as SIRT1, HSP32, CAT, GCL, γGT, and reduce oxidative stress damage (Hu et al., 2017; Xu et al., 2014; Huang et al., 2014). Consequently, HBO is increasingly recognized for its role in injury prevention as a pretreatment measure. Studies have shown that HBO pretreatment effectively reduces VEC injury caused by inflammation, ischemia, and hypoxia by upregulating the expression of HSP27, HSP90, SOD, CAT, BCL-2, and so on. Our preliminary research found that HBO pretreatment significantly alleviates DCS injury and concurrently reduces VEC-related oxidative and inflammatory damage (Fan et al., 2010; Qing et al., 2017). These findings indicate the potential of HBO pretreatment in mitigating DCS-induced VEC damage.

However, the precise mechanisms by which HBO pretreatment mitigates bubble-induced VEC injury remain unclear. Our previous studies identified the induction of heat shock protein (HSP) as a crucial mechanism underlying the protective effects of HBO pretreatment against DCS (Ni et al., 2013; Magee et al., 1994). In a study on Bama pigs with DCS, we observed a significant increase in the expression of lymphocyte HSPs (32, 60, 90) after HBO exposure (Schottlender et al., 2021). HSPs are proteins that respond to heat stress and exert protective and reparative effects, and they are primarily regulated by the transcription factor heat shock factor 1 (HSF-1). Stimuli such as oxidative stress, ischemia, hypoxia, and infection can also activate HSF-1 to upregulate the expression of HSPs. To elucidate the mechanisms underlying HBO pretreatment, we conducted a transcriptomic analysis to investigate gene transcription in pulmonary microvascular endothelial cells (PMVECs), a critical target cell, after HBO. The results revealed a marked increase in the mRNA levels of genes encoding HSP27 and HSP40, both downstream of HSF-1 (Supplementary Figure S1).

Therefore, this study utilized Sprague–Dawley rats and PMVECs to investigate the effects and mechanisms of HBO pretreatment on HSF-1 activation and HSF-1 downstream proteins in DCS-induced VEC injuries, both *in vivo* and *in vitro*. Such discoveries advance our understanding of HBO pretreatment as a potential strategy for DCS prevention.

## Materials and methods

### Cell culture

Rat PMVECs were isolated following a previously described method with modifications (Yu et al., 2017). Male SD rat (weighing 70 ± 10 g) lung tissues were isolated, cut into small pieces, and digested with Trypsin-EDTA (0.25%). The resulting cells were cultured in endothelial cell medium (ECM), with non-adherent cells(e.g., blood cells, dead/dying cells) and debris removed removed at 12 h Incubated until they formed a layer. The cell culture was then transferred to a new dish for further growth. The purity of VEC was validated by detecting von Willebrand Factor (vWF).

### Animals

Male Sprague–Dawley (SD) rats weighing 300 ± 10 g from the Experimental Animal Center of Naval Medical University were used in this study. All rats were housed under controlled temperature (25°C ± 1°C) and relative humidity (54% ± 2%) with 12/12-h light/dark cycle and provided free access to pelleted rodent diet and water. KRIBB11 (30 mg/kg/day) was administered via intraperitoneal injection to rats once daily for three consecutive days prior to HBO treatment. The drug was dissolved in sterile physiological saline containing 10% dimethyl sulfoxide (DMSO) to ensure solubility and stability, and the solution was filtered through a 0.22-μm membrane prior to administration.

All experiments were approved by the Animal Care and Use Committee of the Naval Medical University.

### HBO pretreatment

HBO pretreatment was conducted in controlled environments for the *in vitro* and *in vivo* studies. *In vitro*, PMVECs were exposed to 280 kPa for 60 min in a hyperbaric incubator (OxyCure 3000, OxyHealH Health Group, United States), with rapid 3 min compression and decompression, and pure oxygen with 1.79% CO_2_ to maintain physiological pH. *In vivo*, rodents were subjected to the same pressure duration in a hyperbaric incubator (Type RDC 150-300–6, Naval Medical University, Shanghai, China), with CO_2_ absorbent and continuous oxygen ventilation to prevent CO_2_ buildup, all at a room temperature of 25°C ± 1°C. Absolute pressures were used throughout the study.

### Cell bubble contact injury modeling

A cell bubble contact injury model was established as previously described (Yu et al., 2017). Briefly, PMVECs were inoculated into 35 mm dishes filled with Dulbecco’s modified Eagle’s medium containing 20% fetal bovine serum, sealed, inverted, and then injected with the bubble syringe at 0.5 mL/min into 2 mm bubbles so that the bubbles touched the adherently growing PMVECs for 3 h. After contact, the culture medium was collected and replaced with complete ECM.

### DCS modeling

In the DCS and HBO pretreatment groups, the rats were placed in a transparent hyperbaric rodent chamber (Model RDC150-300-6, Second Military Medical University, Shanghai, China) and compressed with air. The pressure was gradually increased to 7 ATA over 5 min, starting at a low rate of 0.5 ATA/min, to minimize potential middle ear discomfort. This pressure was maintained for 90 min. Subsequently, decompression was performed linearly to ambient pressure at a rate of 2 ATA/min. Throughout the exposure period, the chamber was continuously ventilated with compressed air to prevent CO_2_ retention. DCS was diagnosed using a standardized activity level, whereby rats walked at a rate of 3 m/min for 30 min within an animal transfer cage. The diagnosis of DCS was based on the following symptoms: difficulty breathing, difficulty walking, limb paralysis, convulsions, or death. The occurrence of any one of these symptoms is sufficient to diagnose DCS (Yu et al., 2017).

### Western blot

Cytoplasmic and nuclear proteins were extracted using a nuclear and cytoplasmic protein extraction kit (Beyotime, Shanghai, China) following the manufacturer’s instructions. Total proteins were harvested and lysed in RIPA buffer (Beyotime). The samples were then electrophoresed on 10% SDS-polyacrylamide gels, transferred to a PVDF membrane (Millipore, United States), and probed with HSF-1(4356S, 1:1000), HSP27(95357S, 1:1000), HSP40(4868S, 1:1000), p38 MAPK(9212S, 1:1000), p-p38 MAPK(4511S, 1:1000), ERK1/2(9102S, 1:1000), p-ERK1/2(4370S, 1:2000), MEK1/2(9122S, 1:1000), p-MEK1/2(9154S, 1:1000), Lamin B1(13435S, 1:1000)1, and β-actin (4967S, 1:1000) antibodies (Cell Signaling Technology, CST). Goat anti-rabbit IgG (7074P2, 1:3000) (CST) was used as the secondary antibody. The band intensities were quantified using the Kodak Digital Science 1D Image Analysis System (Eastman Kodak, United States).

### Immunofluorescence staining

To verify the expression, the cultures on PLL-coated coverslips were fixed with 4% paraformaldehyde for 30 min, blocked with 5% BSA for 20 min at room temperature, and incubated with HSF-1 (95357S, 1:500), HSP27(95357S, 1:400), and HSP40(4868S, 1:4000) primary antibodies (CST) overnight at 4°C. The samples were washed three times with TBST and then treated with a fluorescein isothiocyanate (FITC)/Cy3-conjugated anti-rabbit secondary antibody (Proteintech, China) at room temperature for 1 h. Then, the nuclei were stained with 4′,6-diamidino-2-phenylindole (DAPI) working solution (Beyotime) for 10 min, with the FITC-conjugated goat anti-rabbit IgG (39572S, 1:500) (CST) for 1 h at 37°C, and mounted with Gel/Mount aqueous mounting media containing DAPI. Then, the cultures were observed under a fluorescence microscope (DMi8; Leica Camera, Germany).

### Enzyme-linked immunosorbent assay (ELISA)

The cultural solutions or rat venous serum were collected and centrifuged at 1500 *g* at 4°C for 15 min. The supernatant was collected and stored at −80°C for further study. Soluble ICAM-1/ET-1/TNF-α/Il-β (Jiancheng Bioengineering Institute, Nanjing, China) were determined in accordance with the manufacturer’s instructions.

### Flow cytometry analysis

Apoptosis of PMVECs was detected using the annexin V-propidium iodide (PI) apoptosis detection kit (Beyotime). Briefly, 1 × 10^5^/test of PMVECs were resuspended in 500 μL of binding buffer and then labeled with annexin V-FITC (5 μL) and PI (5 μL) for 15 min in the dark at room temperature. Annexin V-FITC and PI were examined with a FACScan flow cytometer (Becton-Dickinson Biosciences, San Jose, CA). The examination wavelength was 488 nm, and the emission wavelength was 530 nm. Endothelial microparticles (EMPs) were generated and detected following a previously described method by Yu et al. (Bove, 2014) with modifications. EMPs were incubated with annexin V-FITC for analysis, whereas EMPs from the rats were double-stained with annexin V-FITC and anti-CD144-PE (Santa Cruz). The EMPs were analyzed using a FACScan flow cytometer (Becton-Dickinson Biosciences).

### Hematoxylin and eosin (H&E) staining and lung injury score

The lungs were fixed in 4% paraformaldehyde, embedded in paraffin, cut into 5 μM sections, and subjected to H&E staining in accordance with routine histopathological methods. Histopathological changes were observed using a light microscope (ZEISS, Germany).

### Reactive oxygen species (ROS) detection

Levels of intracellular ROS were measured using the mitochondria-targeted ROS fluorescent probe Mito-Sox (Thermo Fisher Scientific). PMVECs were incubated with Mito-Sox (5 μM) for 30 min at 37 °C. Fluorescence intensity was observed under a fluorescence microscope at an excitation wavelength of 495 nm and emission wavelength of 515 nm (ZEISS, Germany) immediately after HBO exposure.

### Statistical analysis

All data are presented as mean ± standard deviation (SD). Statistical analyses were performed using one-way ANOVA in SPSS (version 26.0) and Prism (version 9.5.1). Statistical significance was considered at *P* < 0.05.

## Results

### HBO induces HSF-1 activation and HSP expression in PMVECs

**FIGURE 1:**
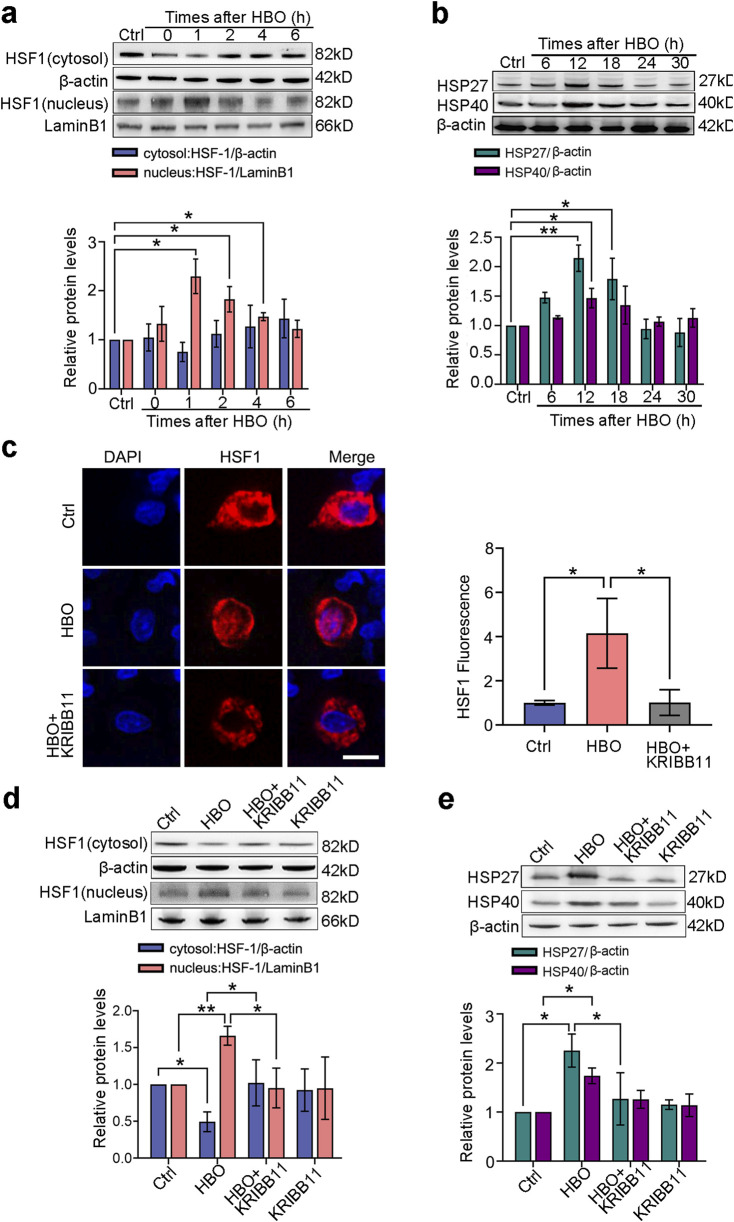
Effects of HBO pretreatment on HSF-1 activation and HSP expression in PMVECs. Cultured PMVECs were exposed to HBO (280 kPa-60 min), and the expression levels of HSF-1 nuclear translocation and downstream proteins were detected by Western blot or *in situ* immunofluorescence staining. HSF-1 inhibitor KRIBB11 (30 μM) was applied 12 h before HBO exposure. **(a)** Nucleus and cytosol levels of HSF-1 protein after HBO; **(b)** Time-dependent cellular HSP27 and HSP40 levels after HBO; **(c)** Subcellular distribution of HSF-1(red), 12 h after HBO, nuclei were labeled with DAPI (blue), scale bar: 50 μm; **(d)** HSF1 nuclear translocation after KRIBB11 treatment; **(e)** HSP27 and HSP40 expression levels after KRIBB11 treatment. HBO: hyperbaric oxygen; HSP: heat shock protein; HSF-1: heat shock factor 1. *n* = 3, *P < 0.05, **P < 0.01.

RNA-seq analysis was conducted to systematically investigate the effects of HBO pretreatment on the protein expression of PMVECs. Results revealed gene transcriptional alterations in PMVECs after HBO exposure. As demonstrated in Supplementary Figure S1a, the transcription levels of 177 genes were significantly upregulated and those of 250 genes were downregulated after HBO exposure. Notably, nine genes downstream of the transcription factor HSF-1 were markedly upregulated, including *hsf1, hspb1*, and *Dnajb1*, which encode HSP27 and HSP40 (Supplementary Figure S1b). Western blot and immunofluorescence confirmed the activation of HSF-1 and expression of HSP27 and HSP40, subsequently. Nuclear translocation of HSF-1 peaked around 1 h after HBO exposure (*P* < 0.01, Figure 1a), whereas HSP27 and HSP40 reached their maximum levels at approximately 12 h after HBO exposure (*P* < 0.05, Figure 1b). Treatment with the HSF-1 inhibitor KRIBB11 inhibited HSF-1 activation (Figures 1c,d) and antagonized HSP27 and HSP40 upregulation induced by HBO (*P* < 0.05, Figure 1e).

### HBO pretreatment counteracts bubble-induced PMVEC injury via HSF-1

**FIGURE 2:**
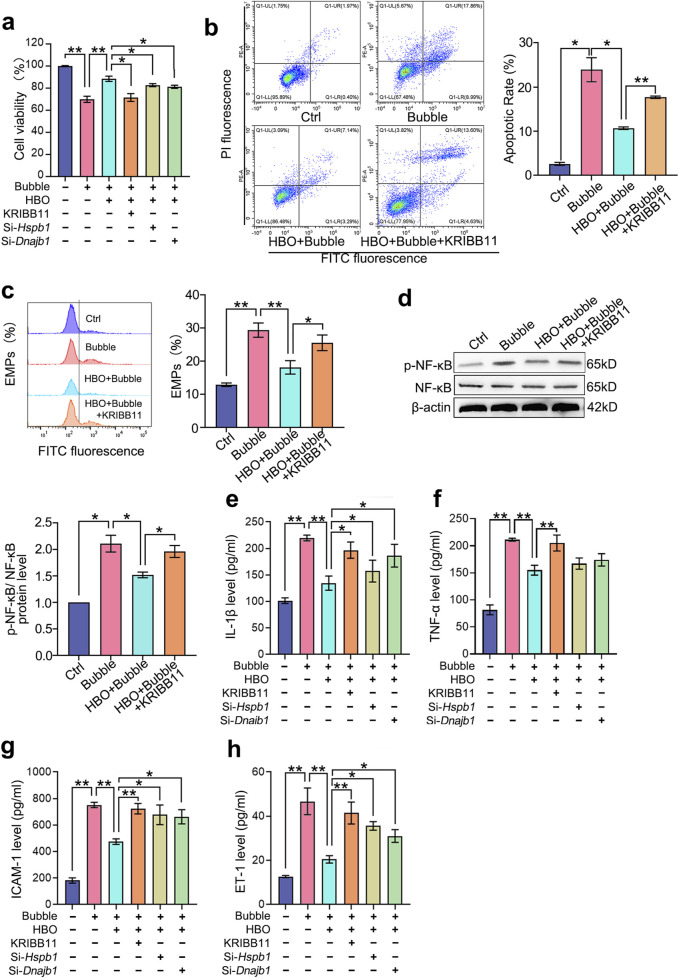
Roles of HSF-1, HSP27, and HSP40 in HBO pretreatment against bubble-induced PMVEC injury. Cultured PMVECs were exposed to bubble contact after HBO or not. HSF-1 activation was inhibited by KRIBB11, and the expression of HSP27 and HSP40 was interfered by si-*hspb1* and si-*Dnajb1* respectively, before HBO exposure. **(a)** Cell viability (*n* = 8); **(b)** Cell apoptosis (*n* = 3); **(c)** EMPs content in supernatant (*n* = 3); **(d)** NF-κB phosphorylation (*n* = 3); **(e)** IL-1β, **(f)** TNF-α, **(g)** ICAM-1, **(h)** ET-1 content in supernatant (*n* = 8). EMPs: endothelial microparticles. *P < 0.05, **P < 0.01.

Compared with the control group, the bubble-contacted group had significantly lower PMVEC cell viability (*P* < 0.01, Figure 2a) and significantly higher apoptosis levels (*P* < 0.05, Figure 2b), NF-κB phosphorylation (*P* < 0.05, Figure 2d), and EMPs (*P* < 0.01, Figure 2c). Additionally, inflammatory factors IL-1β (*P* < 0.01, Figure 2e) and TNF-α (*P* < 0.01, Figure 2f), as well as endothelial injury markers ICAM-1 (*P* < 0.01, Figure 2g) and ET-1 (*P* < 0.01, Figure 2h) were significantly upregulated. HBO pretreatment significantly mitigated these effects of bubble contact. However, inhibiting HSF-1 activation with KRIBB11 and suppressing HSP27 and HSP40 expression with si-hspb1 and si-Dnajb1 significantly counteracted the protective effects of HBO pretreatment against bubble-induced injury. This result suggests that HBO pretreatment alleviates the damage to PMVECs caused by air bubbles by modulating HSF-1, HSP27, and HSP40.

### HBO activates HSF-1 in PMVECs via the mitochondrial ROS/AKT pathway

**FIGURE 3:**
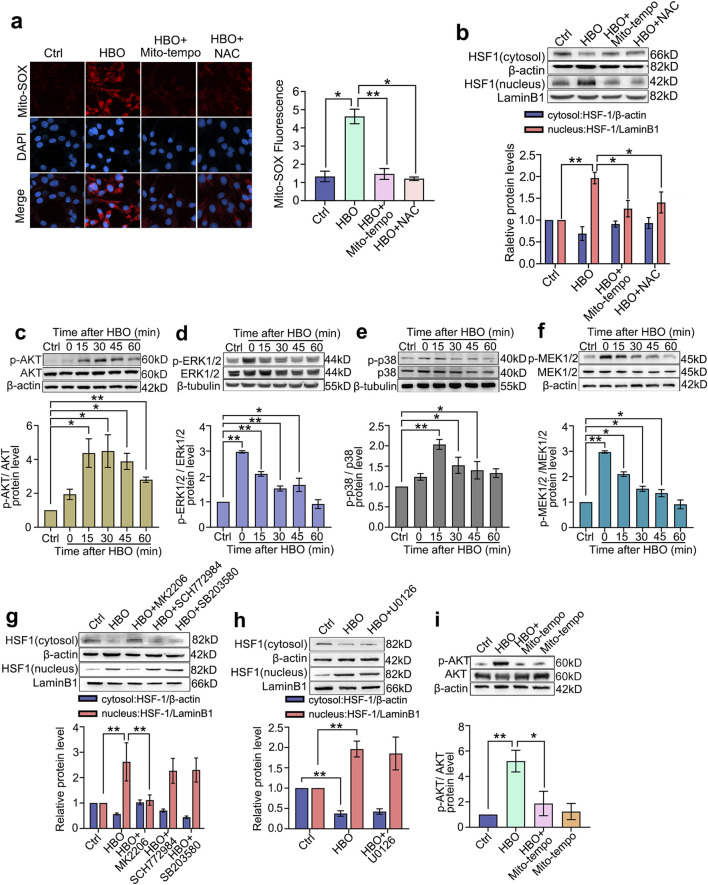
HBO Pretreatment Activates the AKT/HSF-1 Pathway via ROS Elevation. ROS were labeled by mitochondrial-targeted fluorescent probe Mito-SOX during HBO exposure. Mitochondrial-targeted ROS scavenger Mito-Tempo and whole-cellular ROS scavenger NAC were applied 1 h before HBO. **(a)** Fluorescence staining of mitochondrial ROS (Red), nuclei were marked with DAPI (Blue) (scale bar: 20 μm); **(b)** HSF-1 nuclear translocation. ROS: reactive oxygen species. *n* = 3, *P < 0.05, **P < 0.01.Western blot was used to detect the activation of signaling molecules. Specific inhibitors or ROS scavengers were applied before HBO exposure. **(c–f)** Phosphorylation levels of Akt, ERK1/2, p38 MAPK and MEK1/2 after HBO exposure; **(g–h)** HSF-1 nuclear translocation after inhibition of ROS or signaling molecules. **(i)** AKT phosphorylation after ROS elimination. *n* = 3, *P < 0.05, **P < 0.01.

Mito-SOX results showed that the mitochondrial ROS levels in PMVECs significantly increased after HBO exposure (*P* < 0.05, Figure 3a). Both mitochondrial (Mito-Tempo) and whole-cell (NAC) ROS elimination inhibited HSF-1 nuclear translocation (*P* < 0.05, Figure 3b). This result suggests a mitochondrial ROS-dependent HSF-1 activation after HBO exposure. HBO exposure significantly increased the phosphorylation of signaling molecules, such as AKT, ERK1/2, p38 MAPK, and MEK1/2(*P* < 0.05, Figures 3c–f). The phosphorylation levels of ERK1/2and MEK1/2 (*P* < 0.01, Figures 3d–f) peaked immediately after HBO exposure, whereas those of AKT (*P* < 0.05, Figure 3c) and p38 MAPK (*P* < 0.01, Figure 3e) peaked at 30 and 15 min after HBO exposure, respectively. The inhibitory effect of the signal molecule phosphorylation inhibitor is provided in Supplementary Material (Supplementary Figure S2). Inhibition of AKT (MK2206) blocked HSF-1 nuclear translocation (*P* < 0.01, Figure 3g). However, the inhibition of ERK1/2 (SCH772984), p38 MAPK (SB203580), and MEK1/2 (U0126) did not affect HSF-1 activation (Figures 3g,h). The mitochondrial ROS scavenger Mito-Tempo suppressed HBO-induced AKT phosphorylation (*P* < 0.01, Figure 3i), which links ROS to AKT/HSF activation.

### HBO activation of HSF-1 in rat lung promotes HSP27 and HSP40 expression

**FIGURE 4:**
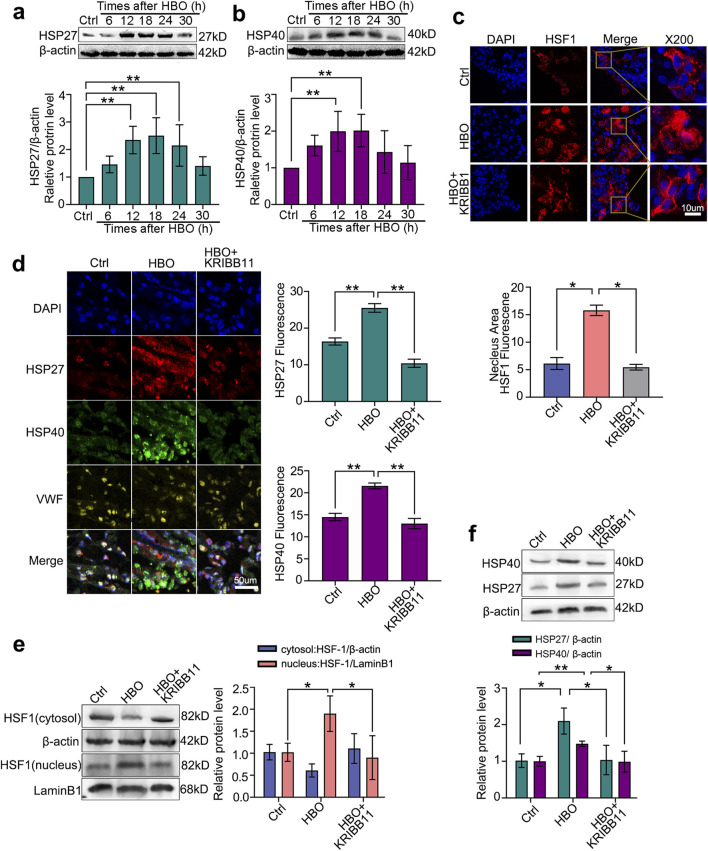
Effects of HBO pretreatment on HSF-1 activation and HSP27/HSP40 expression in rat lung. SD rats received 280 kPa-60 min HBO exposure, and lung tissues were sampled post-HBO. **(a,b)** HSP40 expression after HBO exposure; **(c)** Immunofluorescence staining of HSF-1 (red) in nuclear translocation, cell nucleus stained with DAPI (blue); **(d)** Immunofluorescence staining of HSP27 (green) and HSP40 (red) expression, endothelial cells labeled with vWF (yellow) and nuclei labeled with DAPI (blue); **(e)** HSF-1 nuclear translocation after KRIBB11 treatment; **(f)** HSP27 and HSP40 expression after KRIBB11 treatment. *n* = 6, *P < 0.05,**P < 0.01.

Consistent with the *in vitro* study, HBO exposure significantly increased HSF-1 nuclear translocation (*P* < 0.05, Figure 4c) and elevated HSP27 and HSP40 expression in the rat lungs, peaking approximately 18 h after HBO (*P* < 0.05, Figures 4a,b). Colocalization immunofluorescence of HSF-1, HSP, and vWF confirmed the presence of the HBO/HSF/HSP axis in rat lung VECs (*P* < 0.05, Figure 4d). However, treatment with the HSF-1 inhibitor KRIBB11 reversed these effects (*P* < 0.05, Figures 4c,e,f).

### HBO pretreatment alleviates lung injury via HSF-1 in DCS rats

**FIGURE 5:**
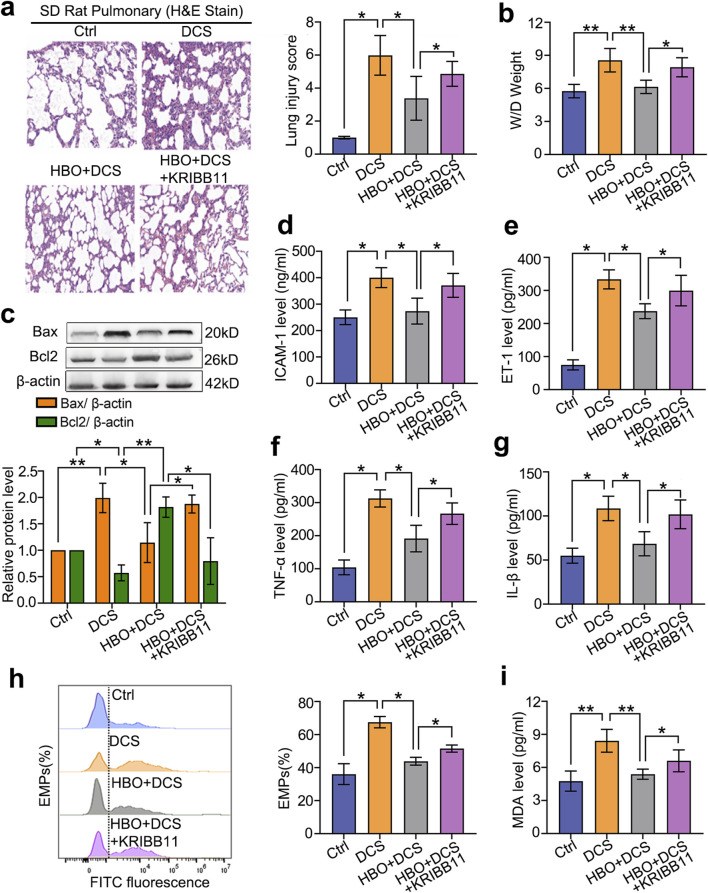
Role of HSF-1 in HBO pretreatment against DCS damage. Rats were treated with the HSF-1 inhibitor KRIBB11 (30 mg/kg) before HBO pretreatment. Subsequently, the DCS model (700 kPa-90 min, 3 min decompression) was established 18 h after HBO pretreatment. **(a)** H&E-stained lung sections; **(b)** lung wet-to-dry ratio; **(c)** apoptotic proteins Bax and Bcl2 levels; **(d–i)** ICAM-1, ET-1, TNF-α, IL-1β, EMP, and MDA contents in serum. *n* = 6, *P < 0.05, **P < 0.01.

**FIGURE 6:**
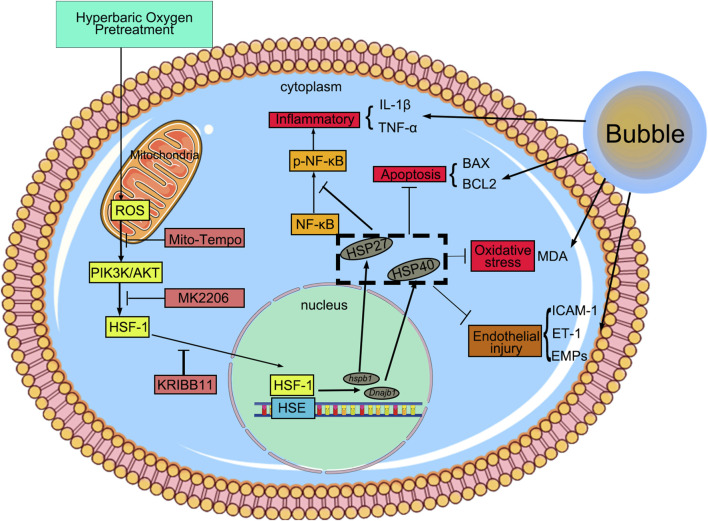
HBO pretreatment reduced DCS/bubble-induced VEC injury via HSF-1. HBO pretreatment triggers mitochondrial ROS in VEC, activating the PIK3K/AKT pathway to promote HSF-1 nuclear translocation. Then, HSF-1 binds to the heat shock element and induces HSP27 and HSP40 expression. This pretreatment exerts anti-inflammatory, anti-apoptotic, and antioxidant effects to reduce bubble-induced VEC injury.

*In vivo*, a rat DCS model was established at the peak expression time of HSPs after HBO exposure. Compared with the control group, DCS modeling significantly increased the lung injury index as the tissue pathological score, wet-to-dry ratio, and apoptotic protein Bax level (*P* < 0.05, *P* < 0.01, Figures 5a–c). In serum, DCS modeling markedly increased endothelial injury markers ICAM-1, ET-1, and EMPs; inflammatory factors TNF-α and IL-1β; and oxidative damage indicator Malondialdehyde (MDA) (*P* < 0.05, Figures 6d–i). HBO pretreatment notably reduced these injury indicators (*P* < 0.05, *P* < 0.01, Figures 5a–i). KRIBB11 abolished the protective effects of HBO (*P* < 0.05, Figures 5a–i), confirming HSF-1 dependency.

## Discussion

VEC injury caused by bubbles is a critical pathogenic mechanism in DCS. During decompression, bubbles form in the venous system, accumulate in the right heart, and are propelled into pulmonary microvessels as microbubbles. These microbubbles eventually dissolve and are expelled via alveolar gas exchange (Shen et al., 2023). The narrow diameter of pulmonary microvessels facilitates direct contact between the bubbles and VECs, leading to mechanical and biochemical endothelial injury. PMVEC damage can lead to pathological processes such as pulmonary edema and inflammatory activation, contributing to DCS pulmonary injury (Vihervaara and Sistonen, 2014). Our previous study showed that bubble contact triggers PMVECs to release endothelial microparticles, promoting DCS by initiating inflammatory cascades and inducing VEC apoptosis (Bove, 2014). *In vivo* and *in vitro* studies further confirmed that HBO pretreatment enhances PMVEC resistance to bubble injury by activating HSF-1 and upregulating downstream proteins, thereby alleviating DCS damage.

HSF-1 is a crucial transcription factor that is involved in various stress responses (Aolymat et al., 2023). Under normal conditions, HSF-1 exists in its inactive polymeric form in the cytoplasm (Gomez-Pastor et al., 2018). Upon exposure to heat shock or oxidative stress, HSF-1 dissociates from the complex, translocates to the nucleus, binds to the heat shock element, and activates HSP gene expression to exert protective effects (Janowska et al., 2019). In the present study, we first observed the nuclear translocation of HSF-1 and verified its downstream protein expression using Western blot analysis after HBO exposure. The results showed a significant increase in HSF-1 nuclear translocation, which peaked 1 h after HBO exposure. A previous transcriptomic analysis indicated that the transcription levels of Hspb1 and Dnajb1, which encode HSP27 and HSP40 proteins, respectively, are significantly elevated. Western blot validation confirmed the upregulation of these proteins after HBO exposure, peaking at 12 h. At the peak expression time points of HSP27 and HSP40, we established a PMVEC bubble injury model to investigate their roles in HBO pretreatment-mediated protection. The results demonstrated that HBO pretreatment significantly reduced bubble-induced apoptosis, inflammatory responses, and endothelial microparticle release, thereby enhancing the resistance of PMVECs to bubble injury. Inhibition of HSF-1, HSP27, or HSP40 function using specific inhibitors or siRNA significantly antagonized the protective effects of HBO pretreatment, suggesting that HBO pretreatment protects PMVECs against bubble injury by activating HSF-1 and upregulating HSP27 and HSP40 expression.

HSP27, a small heat shock protein, acts as a molecular chaperone by binding to cytoskeletal proteins to maintain cell morphology and structural stability while participating in cell proliferation, migration, and inhibiting apoptosis (Kampi et al., 2010). HSP40, a J-domain-containing member of the heat shock protein family, primarily functions as a cofactor of HSP70, enhancing its ATPase activity to facilitate proper protein folding and reduce cellular damage (Horng et al., 2001). Additionally, HSP40 can activate Toll-like receptors to combat infection (Liu X. et al., 2022), bind to denatured/damaged proteins to aid refolding (Chien et al., 2010), and interact with BIP to maintain endoplasmic reticulum (ER) stability and delay amyloid formation (Liu J-F. et al., 2022). In our study, because HSP70 expression was not significantly elevated after HBO exposure, HSP40 may exert its protective effects independent of HSP70.

Under oxidative stress, HSF-1 activity is regulated by the ERK1/2, p38 MAPK, and PI3K/AKT signaling pathways (Tang et al., 2015; Chen et al., 2024). To elucidate the mechanism by which HBO exposure activates HSF-1, we measured the ROS levels and phosphorylation of these signaling molecules in PMVECs. The results showed that HBO exposure significantly increased the levels of ROS and phosphorylation of ERK1/2, p38 MAPK, and AKT after HBO exposure. Inhibition of these pathways revealed that AKT inhibition significantly suppressed the HSF-1 activation and HSP27 and HSP40 expression induced by HBO exposure. Although ERK1/2 is a downstream molecule of MEK1/2, our previous studies found that HBO pretreatment upregulates HSP32 expression in cultured spinal neurons through the P38 MAPK/Nrf2 pathway while simultaneously inhibiting excessive HSP32 expression via a MEK1/2-mediated negative regulatory pathway, independent of ERK1/2 (Ni et al., 2013). Therefore, we further investigated the role of MEK1/2 in HBO pretreatment-induced HSF-1 activation. Unlike in cultured spinal neurons, MEK1/2 did not participate in HBO pretreatment-induced HSF-1 activation in our study, suggesting that the mechanisms by which HBO pretreatment upregulates protective proteins may differ among cell types. Considering that ROS generation after HBO exposure primarily originates from mitochondrial pathways, we used the mitochondrial ROS scavenger Mito-Tempo to investigate the role of ROS in AKT activation. The results indicated that AKT activation was significantly inhibited by Mito-Tempo, suggesting that HBO pretreatment may activate HSF-1 and upregulate HSP27 and HSP40 expression through the ROS/AKT signaling pathway.

After confirming the protective effects and mechanisms of HBO pretreatment on PMVECs *in vitro*, we further validated these findings *in vivo*. Western blot and immunohistochemical analyses showed significant activation of HSF-1 and upregulation of HSP27 and HSP40 in rat lung VECs 18 h after HBO exposure, which is consistent with our previous findings where peak protective protein expression occurred around 12 h *in vitro* and 18 h *in vivo* (Ni et al., 2013; Huang et al., 2014). At the peak protein expression time point, we established a rat DCS model and evaluated the protective effects of these proteins. The results demonstrated that DCS caused significant VEC injury, inflammatory cell infiltration, interstitial edema, and alveolar structural damage in rat lungs, which were significantly alleviated by HBO pretreatment. Although specific inhibitors of HSP27 and HSP40 were not available *in vivo*, the protective effect of HBO pretreatment against rat DCS was significantly reversed by the HSF-1 inhibitor KRIBB11, further validating the *in vitro* findings. The lung wet-to-dry weight ratio reflects the degree of inflammatory exudation and interstitial edema in pulmonary tissues (Zhang et al., 2017; Yu et al., 2021). In this study, lung wet-to-dry ratio was significantly elevated in DCS rats, while decreased in HBO pretreatment group indicated that HBO pretreatment alleviated the inflammatory exudation and interstitial edema in pulmonary tissues. The underlying mechanisms may involve:(1) Anti-apoptotic effects mediated by HSF-1-dependent upregulation of anti-apoptotic proteins (e.g., Bcl-2), thereby attenuating cellular apoptosis; (2) Anti-inflammatory actions achieved through HSF-1-mediated suppression of NF-κB activity, which reduces pro-inflammatory cytokine production (e.g., IL-1β, TNF-α) and mitigates systemic inflammatory responses.

Considering that bubble-induced VEC injury is systemic, we also measured the serum levels of ICAM-1, ET-1, EMPs, MDA, TNF-α, and IL-1β as markers of VEC injury. Our previous studies identified ICAM-1 and ET-1 as sensitive indicators of DCS VEC injury, whereas EMPs are released from damaged VECs and reflect VEC injury. TNF-α, IL-1β, and MDA reflect the extent of inflammation and oxidative stress, respectively (Bove, 2014). The results showed significant increases in these markers in the DCS model group, which were effectively ameliorated by pretreatment with HBO. This protective effect was also reversed by KRIBB11, suggesting that HBO protects VECs by activating HSF-1 and may reduce inflammation and oxidative stress.

## Conclusion

HBO pretreatment enhances the resistance of VECs to bubble-induced injury by increasing intracellular ROS levels, activating the AKT/HSF-1 signaling pathway, and upregulating the expression of HSP27 and HSP40. These mechanisms collectively mitigate DCS-related injury in a rat model.

## Data Availability

The original contributions presented in the study are included in the article/Supplementary Material, further inquiries can be directed to the corresponding authors.

## Supplementary material

SUPPLEMENTARY FIGURE S1
Differential Gene Expression Analysis in PMVECs Following HBO Exposure. RNA-seq reveals transcriptomic changes in PMVECs following HBO pretreatment (280 kPa, 60 min). **(a)** Volcano plot of RNA-seq data comparing transcriptional profiles between the Ctrl and HBO groups post-exposure. **(b)** Heatmap of the top 20 differentially expressed genes identified by RNA-seq.Data are expressed as mean ± SD, *n* = 3.

SUPPLEMENTARY FIGURE S2
Evaluation of signal molecule inhibitor efficacy following HBO treatment. PMVECs were treated with MK2206, SCH772984, SB203580 or U0126 followed by HBO exposure. **(a–d)** Western blot analysis was performed to assess the phosphorylation levels of AKT, ERK1/2, p38 MAPK, and MEK1/2. *n* = 3, **P* < 0.05,***P* < 0.01.

## Abbreviations

CCK-8, Cell counting kit-8; DAPI, 4,6-Diamidino-2-phenylindole; DCS, Decompression sickness; EMPs, Endothelial microparticles; ERK, Extracellular signal‐regulated kinases; ET-1, Endothelin-1; FITC, Fluorescein isothiocyanate; HBO, Hyperbaric oxygen; HSF-1, Heat shock factor 1; HSP, Heat shock protein; ICAM-1, Intercellular cell adhesion molecule-1; IL-1β, Interleukin-1β; MDA, Malondialdehyde; NF-κB, Nuclear factor kappa-B; PMVEC, Pulmonary microvascular endothelial cells; ROS, Reactive oxygen species; siRNA, Small interfering RNA; TNF-α, Tumor necrosis factor-α; VEC, Vascular endothelial cell; HSP27, Heat shock protein 27; HSP40, Heat shock protein 40; AKT, Serine/threonine kinase.
