# Supplementary figures and images for: Hyperbaric oxygen pretreatment on endothelial cell injury via heat shock factor 1 in decompression sickness
Source: Front Mol Biosci. 2025 Jun 13;12:1617318. doi: 10.3389/fmolb.2025.1617318 (PMC12202230; doi:10.3389/fmolb.2025.1617318)

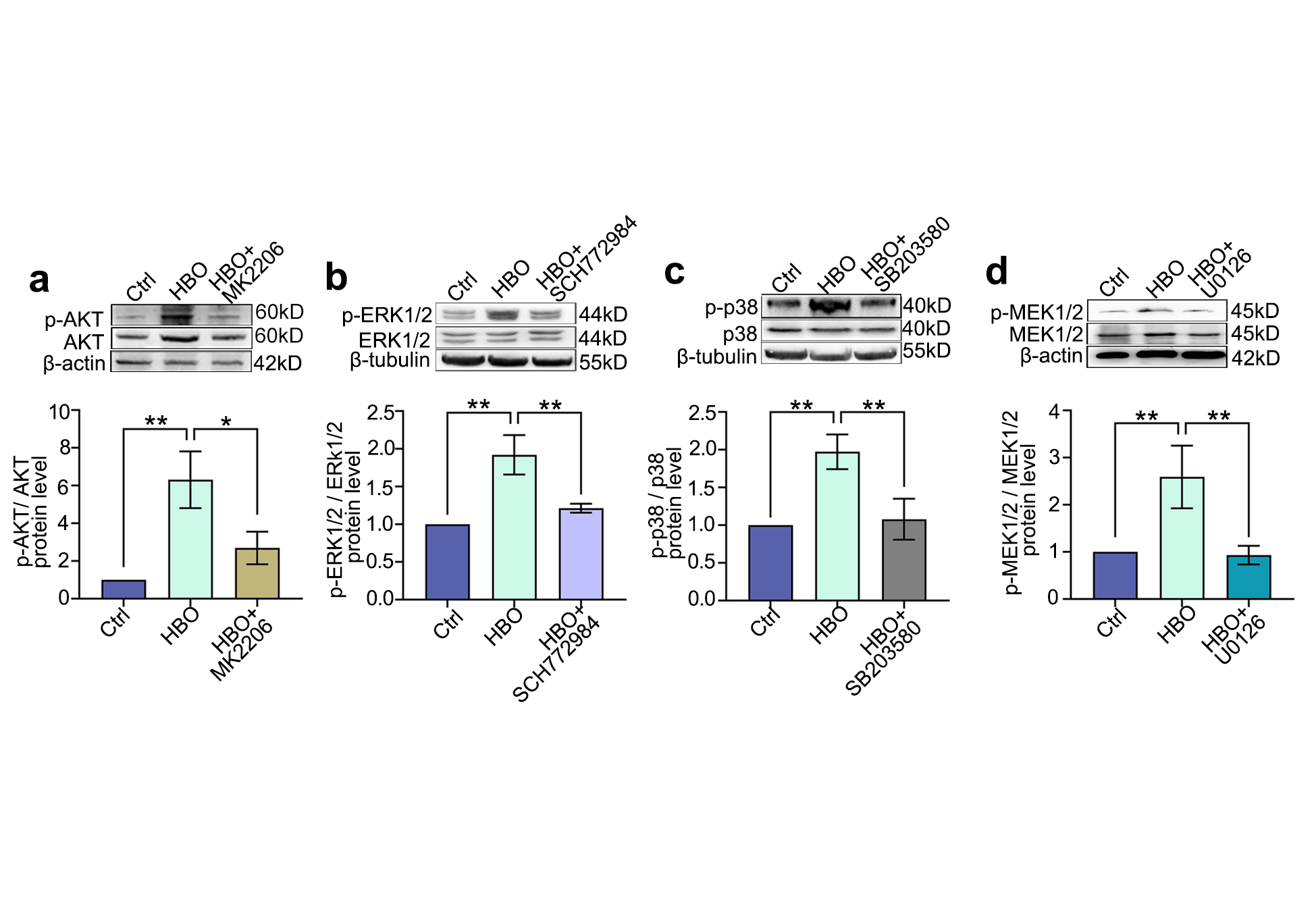

Supplement: Supplementary file 1 [file Image2.tif]

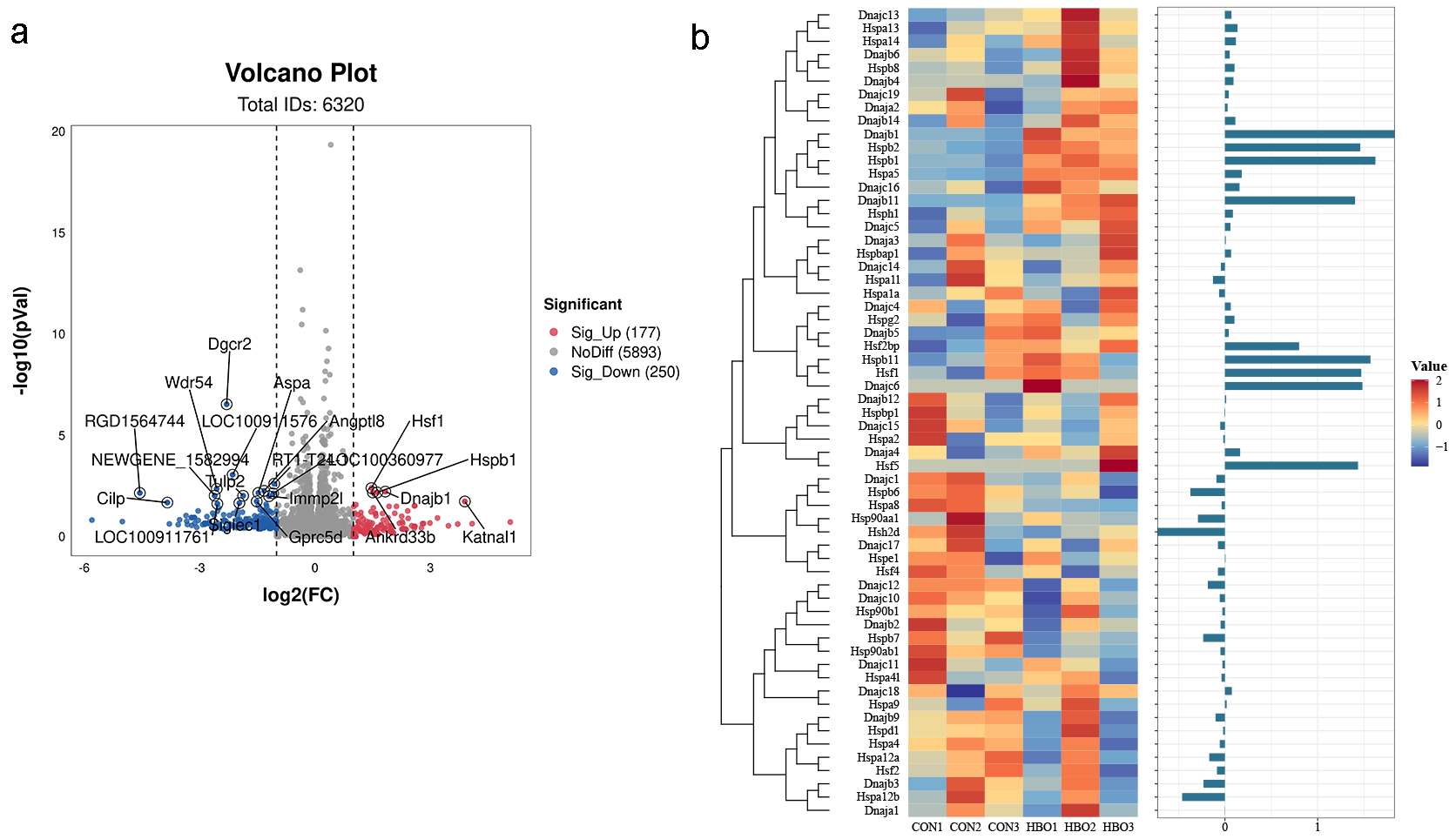

Supplement: Supplementary file 2 [file Image1.tif]
